# Sensitivity and specificity of an electronic nose in diagnosing pulmonary tuberculosis among patients with suspected tuberculosis

**DOI:** 10.1371/journal.pone.0217963

**Published:** 2019-06-13

**Authors:** Antonia M. I. Saktiawati, Ymkje Stienstra, Yanri W. Subronto, Ning Rintiswati, Sumardi, Jan-Willem Gerritsen, Henny Oord, Onno W. Akkerman, Tjip S. van der Werf

**Affiliations:** 1 Department of Internal Medicine, Faculty of Medicine, Public Health and Nursing, Universitas Gadjah Mada, Yogyakarta, Indonesia; 2 University of Groningen, University Medical Center Groningen, Department of Pulmonary Diseases and Tuberculosis, Groningen, the Netherlands; 3 Center for Tropical Medicine, Universitas Gadjah Mada, Yogyakarta, Indonesia; 4 University of Groningen, University Medical Center Groningen, Department of Internal Medicine—Infectious Diseases, Groningen, the Netherlands; 5 Department of Microbiology, Faculty of Medicine, Public Health and Nursing, Universitas Gadjah Mada, Yogyakarta, Indonesia; 6 eNose B.V. (The eNose Company), Zutphen, The Netherlands; 7 University of Groningen, University Medical Center Groningen, Tuberculosis Center Beatrixoord, Haren, the Netherlands; University of Cape Town, SOUTH AFRICA

## Abstract

**Objective:**

To investigate the potency of a hand-held point-of-care electronic-nose to diagnose pulmonary tuberculosis (PTB) among those suspected of PTB.

**Methods:**

Setting: Lung clinics and Dr. Sardjito Hospital, Yogyakarta, Indonesia. Participants: patients with suspected PTB and healthy controls. Sampling: 5 minutes exhaled breath. Sputum-smear-microscopy, culture, chest-radiography, and follow-up for 1.5–2.5 years, were used to classify patients with suspected PTB as active PTB, probably active PTB, probably no PTB, and no PTB. After building a breath model based on active PTB, no PTB, and healthy controls (Calibration phase), we validated the model in all patients with suspected PTB (Validation phase). In each variable (sex, age, Body Mass Index, co-morbidities, smoking status, consumption of alcohol, use of antibiotics, flu symptoms, stress, food and drink intake), one stratum’s Receiver Operating Characteristic (ROC)-curve indicating sensitivity and specificity of the breath test was compared with another stratum’s ROC-curve. Differences between Area-under-the-Curve between strata (*p*<0.05) indicated an association between the variable and sensitivity—specificity of the breath test. Statistical analysis was performed using STATA/SE 15.

**Results:**

Of 400 enrolled participants, 73 were excluded due to extra-pulmonary TB, incomplete data, previous TB, and cancer. Calibration phase involved 182 subjects, and the result was validated in 287 subjects. Sensitivity was 85% (95%CI: 75–92%) and 78% (95%CI: 70–85%), specificity was 55% (95%CI: 44–65%) and 42% (95%CI: 34–50%), in calibration and validation phases, respectively. Test sensitivity and specificity were lower in men.

**Conclusion:**

The electronic-nose showed modest sensitivity and low specificity among patients with suspected PTB. To improve the sensitivity, a larger calibration group needs to be involved. With its portable form, it could be used for TB screening in remote rural areas and health care settings.

## Introduction

On 26 September, 2018, the United Nations (UN) had a high-level meeting in the UN headquarters in New York on tuberculosis (TB). The discussion focused on accelerating actions to end TB by 2030 [1]. In 2017, the death toll was still huge–with 1.3 Million death, and an additional 300 000 among HIV-coinfected, TB leads the causes of death by an infectious disease; in 2017 alone, 10 Million people fell ill with TB [1]. Clearly, new diagnostic tools are needed to identify individuals in the community–and in health care facilities–that continue to spread this airborne disease. In many TB high-burden countries, pulmonary tuberculosis (PTB) is commonly diagnosed by sputum smear microscopic examination [1]. Sputum microscopy is labor-intensive, and the technique does not differentiate *Mycobacterium tuberculosis* (MTB) from non-tuberculosis mycobacteria [2]. Though sputum culture is considered gold standard, it is problematic in low-resource settings, because it is expensive, time-consuming, and vulnerable to technical failure [3]. Nucleic acid amplification techniques such as Xpert MTB/ RIF allows for fast identification of MTB [4], but costs remain challenging; it still requires sputum sampling and is neither portable nor fit for point-of-care in remote rural areas with unstable electricity supply. For all of the above-mentioned sputum-based tests, appropriate sputum specimens are required. Meanwhile, not all patients with suspected TB were able to collect an adequate and good quality sputum sample. Chest radiography (CXR), a non-sputum-based test that is usually used, lacks specificity [3].

There is an increasing evidence that analysis of exhaled breath using electronic nose (e-nose) could be used as a novel diagnostic technique [5–9]. An e-nose is a machine that can detect and differentiate odours from any biological materials, such as breath, urine, or faeces, with a sensor array. This array consists of non-specific sensors. An odour stimulates the sensor array to produce a specific fingerprint. Patterns or fingerprints from known odours are used to build a model and train a pattern recognition system, thus unknown odours can be classified based on this model [10]. E-nose has been used for diagnosis of various diseases, *i*.*e*. asthma [5], Chronic Obstructive Pulmonary Disease (COPD) [5,6], urinary tract infection [7], lung cancer [8], and brain cancer [9]. A prototype of an e-nose to diagnose PTB was used by Bruins *et al*. in Bangladesh [11]. They found sensitivity of 93.5% and a specificity of 85.3% to differentiate PTB patients from healthy controls, and a sensitivity of 76.5% and specificity of 87.2% when differentiating PTB patients from other subjects (non-PTB patients and healthy subjects) [11]. However, this e-nose prototype used separate bags to collect exhaled breath that might introduce errors due to interaction between the Volatile Organic Compounds (VOCs) with the bags materials [12]. A newer device that is portable, use rechargeable battery, and does not use separate bags (Aeonose) was tested in Paraguay, and showed high sensitivity (88%) and specificity (92%) to differentiate PTB patients from asthma/COPD and healthy subjects [12].

No previous studies have investigated the potential of the e-nose (Aeonose) device to diagnose PTB among patients presenting with signs and symptoms suggesting PTB, while this would typically provide the added value of such test. We therefore investigated the diagnostic potential of Aeonose to identify PTB among patients with suspected PTB. Our secondary objective was to investigate factors that associated with the sensitivity and specificity of the breath test. The study was conducted in Indonesia, a lower-middle-income country with a population of 264 million, being the third highest TB-burdened country in the world [1]. Many patients live in remote rural areas with difficult access to health care facilities and human resources [13]—a setting, where an accurate and easy-to-handle e-nose would be a tremendous asset.

## Materials and methods

### Study design

In this diagnostic cross-sectional study, we enrolled a cohort of patients with suspected TB and healthy controls. We conducted breath tests, and followed study participants over time to confirm correct diagnosis. Patients with suspected TB were recruited consecutively from the public lung clinics in Yogyakarta and Dr. Sardjito Hospital, Yogyakarta, Indonesia between October 2013 and December 2015. Healthy controls were recruited from neighboring area of subjects who were diagnosed with PTB. Subjects were aged ≥18 years, agreed to participate in the study, were able to produce sputum (except for healthy controls) and exhaled air samples. Healthy controls should have no sign nor symptoms of TB. Subjects were excluded if they had invalid measurements of breath tests, incomplete CXR data, missing sputum specimens (except for healthy controls), incomplete follow-up, or a previous history of PTB or extra-pulmonary TB. Subjects were further excluded from the analysis if they had cancer, because cancer has been known to interfere with the breath prints [8,9]. All subjects followed the same diagnostic work-up.

The study protocol followed the guidelines of the Helsinki Declaration of 2013, was approved by the institutional review board at the Faculty of Medicine, Universitas Gadjah Mada, Yogyakarta, Indonesia (KE/FK/859/EC), and registered at clinicaltrials.gov (NCT02219945). Written informed consent was obtained from each subject before enrollment in the study.

### Test methods

**Fig 1:**
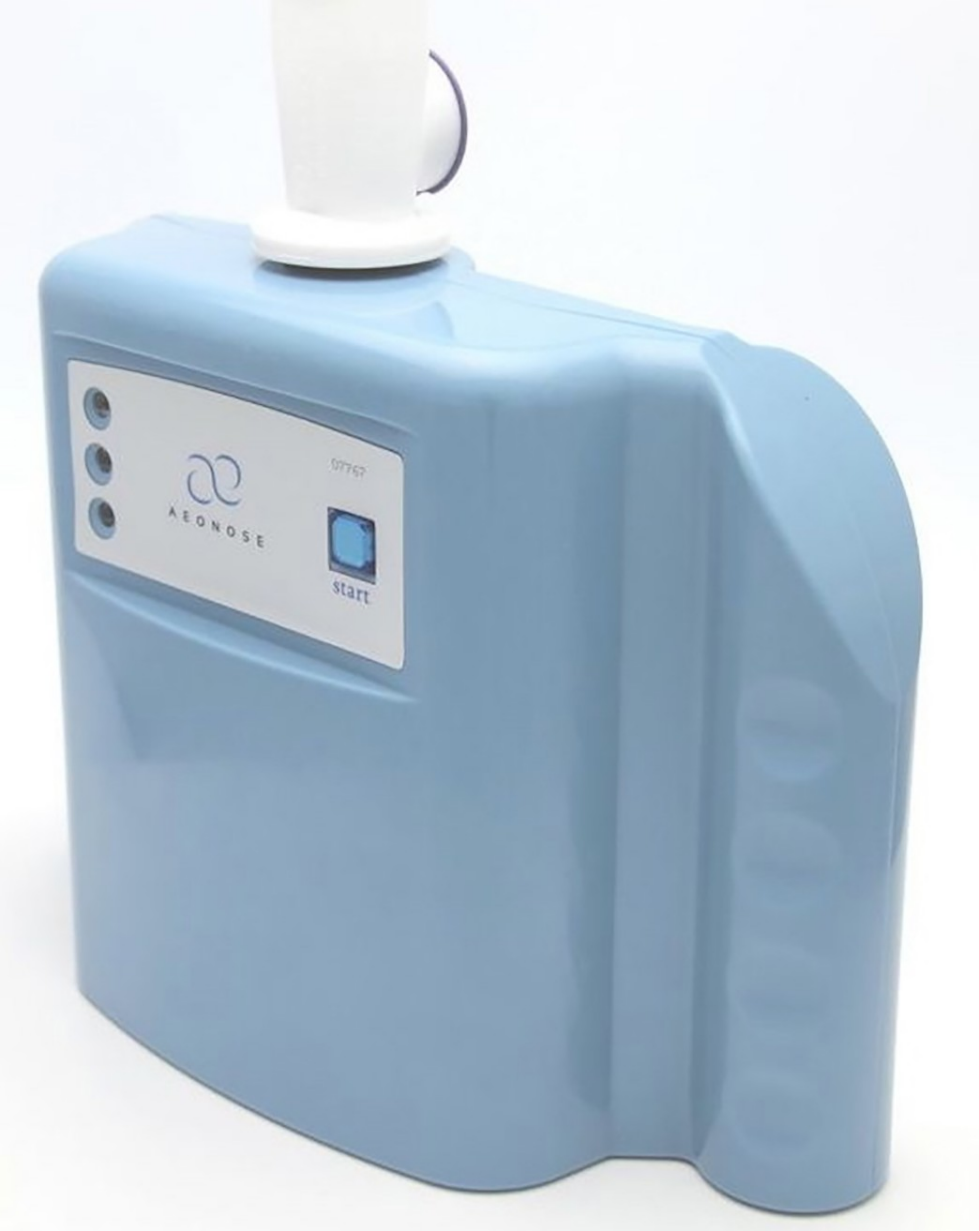
The Aeonose.

The Aeonose (eNose BV, Zutphen, The Netherlands) is an e-nose device combining 3 different metal-oxide sensors (Applied Sensors Gmbh) and a pre-concentrator (Fig 1). A small pump inside the Aeonose ensures that a constant flow of exhaled air passes the three sensor surfaces and a Tenax tube, which enables detecting low concentrations and high boiling Volatile Organic Compounds (VOCs). A 32-step sinusoidal modulation of the sensor surface with temperature between 260–340°C is used to measure the volatile molecular pattern in exhaled air in terms of sensor’s conductivity values. Study participants breathed normally through the Aeonose via a disposable mouthpiece for 5 min. This mouthpiece contains a High Efficiency Particulate Air-filter protecting the Aeonose from getting contaminated by bacteria and viruses; and a valve and carbon filter that prevents interference by Volatile Organic Compounds (VOCs) in the environment that could distort the measurement. A nose clamp was used to prevent non-filtered air from entering the device and to ensure that the total exhaled volume during tidal breathing passed through the device. After measuring the breath, the sensors are regenerated for 10 minutes. The breath data were downloaded into a laptop, and uploaded onto the website of eNose for analysis.

As part of routine examination, all patients were characterized by clinical symptoms (persistent cough, unintentional ≥5% weight loss, and night sweats), three sputum smear microscopic examinations, and CXR. For research purposes, we added sputum culture, HIV testing, and follow-up for 1.5 years after diagnosis. Patients whose culture results disagreed with the initial clinical diagnosis (i.e. culture was positive for MTB, but the clinical diagnosis was non-PTB, or culture was negative despite a clinical diagnosis of PTB) were followed up for 2.5 years. Results of PTB routine examination were available to those seeing the patients during follow-up, but not to the laboratory personnel who processed the culture.

All these data formed a Composite Reference Standard (CRS) [14,15] that classified participants into four categories: active PTB (subjects who scored PTB by all tests), no PTB or Healthy controls (subjects who scored no-PTB by all tests), and patients whose test results classified in between these two extreme groups (Table 1). Follow-up as a part of a CRS has been used successfully in different settings [15–18]. Culture though gold standard for TB but single spot sputum culture is challenging; low quality of sputum samples and laboratory errors may jeopardize both sensitivity and specificity. The CRS that diagnoses TB based on comprehensive results of clinical symptoms, bacteriological examinations, CXR, and follow-up, could address these drawbacks of culture [15,19].

**Table 1:** Composite reference standard used in the study [14–18].

Type of test	Active PTB	Probably active PTB	Probably no PTB	No PTB or Healthy controls
At least 2 out of 3 clinical symptoms suggest PTB	Yes	(a) 4 tests suggest TB including sputum smear; OR (b) 3 tests suggest TB, including culture and/or follow-up; OR (c) 2 tests (culture AND follow-up) suggest TB	(a) 1 test suggest TB; OR (b) 2 tests suggest TB, except culture and/or follow-up; OR (c) 3 tests suggest TB, except culture AND follow-up	No
Sputum smear examination shows Acid Fast Bacilli	Yes/No			No
Culture is positive for *M*. *tuberculosis*	Yes			No
Chest radiography suggests PTB	Yes			No
Follow-up suggests PTB	Yes			No

PTB: pulmonary tuberculosis

Demographic data, smoking history, co-morbidities (Type II diabetes mellitus, HIV, COPD, asthma), presence of flu-like illness, presence of psychological stress due to breath test, co-medication including antibiotics, and food and beverage intake ≤8 hours before the test were recorded.

### Sample analysis

Sputum Ziehl-Neelsen microscopy and culture on Löwenstein-Jensen media followed WHO guidelines [20], in the TB-Microbiology laboratory, Faculty of Medicine Universitas Gadjah Mada. For research purposes, the CXRs read by the attending physicians were re-read by one independent physician (TSW–a pulmonologist). In case of disagreement, the conclusion from the independent reviewer was followed, and the result of this re-reading was used for the CRS.

The breath data were standardized to facilitate measurements between different Aeonose devices. Temperature control of the sensors was key in order to use multiple metal-oxide sensor eNoses in a single study and later on in validation process; when the sensor temperature was kept within narrow limits, sensors with similar material properties showed similar response to VOC’s. When the sensors age, the signal may decrease due to a reduced number of active sites at the sensor surface. Differences between sensors because of aging were removed in data pre-processing by rescaling the distribution of values (subtracting the mean value or centering the data) so that the mean of observed values is 0 and the standard deviation is 1 [21].

The data were compressed with a TUCKER3-solution, and analyzed using an artificial neural network, thus VOC-samples from individuals could be classified as ‘sick’ or ‘healthy’. Results were obtained for different scaling preprocessing methods, seven sensor combinations and several artificial neural network-topologies resulting in multiple models representing the measurement data. The models were presented in Receiver Operating Characteristic (ROC)-curves indicating trade-offs for sensitivity and specificity of breath test in diagnosing PTB, and the best Area Under the Curve (AUC) was selected. Leave-10%-Out cross validation was applied.

We started with a calibration phase to build a breath model, involving participants in “Active PTB”, “no PTB”, and “Healthy control” groups. The e-nose can only classify unknown patients correctly if the patient characteristics (indication, social status, geographical area) are similar to the ones in the calibration set to have fair classification. Therefore, when collecting the breath samples, we kept participants from the “Probably active PTB” and “Probably no PTB” groups blinded for validation purposes. In this validation phase, the blinded breath samples were classified based on the model generated in the calibration phase [22].

We calculated the sensitivity, specificity, and positive and negative predictive values (PPV and NPV) of the breath test using the CRS as the reference standard. To examine the influence of age, participants were divided into 2 groups, based on the median age. In each variable, one stratum’s Receiver Operating Characteristic (ROC)-curve indicating sensitivity and specificity of breath test was compared with another stratum’s ROC-curve. A significant difference of an AUC between strata (*p*<0.05) indicated an association between the variable and sensitivity—specificity of the breath test. Statistical analysis was performed using STATA/SE 15 (License: University of Groningen).

To detect a difference of 15% between the CRS and the breath test with a desired sensitivity of 90%, and assuming a prevalence of TB of 36% among the study subjects based on previous data in the lung clinics (unpublished data), with an α error of 0.05 and a power of 90%, the number of patients with suspected PTB needed for the study was 300 [23]. We followed the STARD guidelines for reporting as appropriate.

## Results

**Fig 2:**
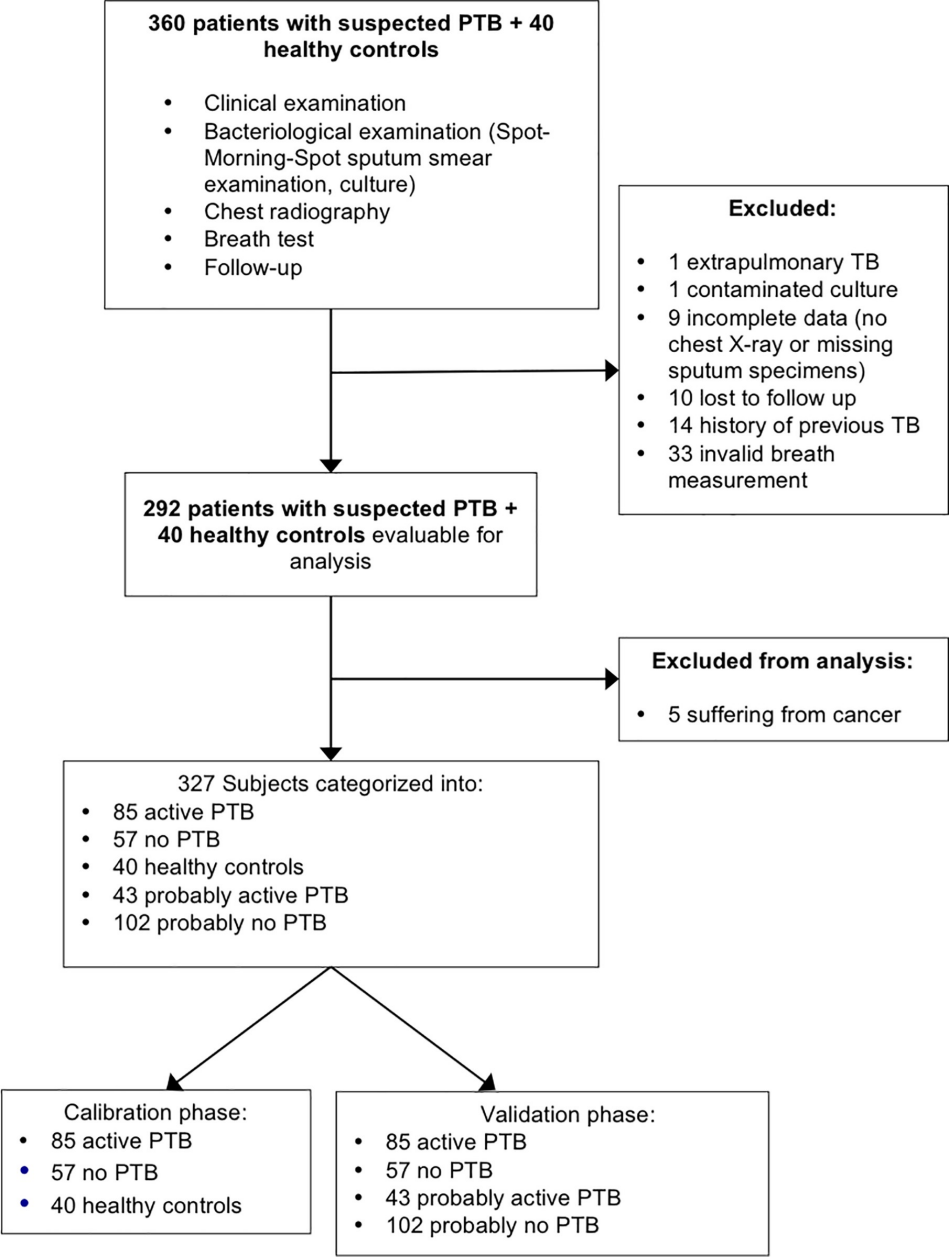
Flow-chart of study participants.

We included 360 consecutive patients with suspected PTB and 40 healthy controls; 73 study participants were excluded for various reasons, resulting in a total of 327 study participants (Fig 2). Table 2 shows that median age of study participants in calibration and validation phase was 40 (range: 18–85) and 46 (range: 18–85) years old, respectively. Diagnoses for patients who turned out to have no PTB included asthma, pneumonia, bronchiectasis, chronic bronchitis, COPD, Obstructive Syndrome Post TB, lung fibrosis, lung abscess, empyema, and polycystic lung disease.

**Table 2:** Characteristics of study participants in each category.

Characteristic					
Calibration phase	Healthy Controls (n = 40)	Active PTB (n = 85)	No PTB (n = 57)		
Validation phase		Active PTB (n = 85)	No PTB (n = 57)	Probably active PTB (n = 43)	Probably no PTB (n = 102)
Sex male, %	60.0	69.4	56.1	60.5	60.8
Age, years median (min-max)	35 (18–66)	38 (19–80)	47 (22–85)	41 (18–77)	52 (19–80)
Body mass index, kg/m^2^ median (min-max)	23.4 (17.2–32.9)	18.1 (14.0–27.9)	20. 9 (13.8–36.7)	18.2 (11.3–25.0)	19.9 (11.9–30.1)
HIV infection, n (%)	0 (0.0)	1 (1.2)	0 (0.0)	3 (7.0)	1 (1.0)
Type II diabetes mellitus, n (%)	1 (2.5)	10 (11.8)	3 (5.3)	6 (14)	4 (3.9)

PTB: pulmonary tuberculosis, HIV: human immunodeficiency virus

**Fig 3:**
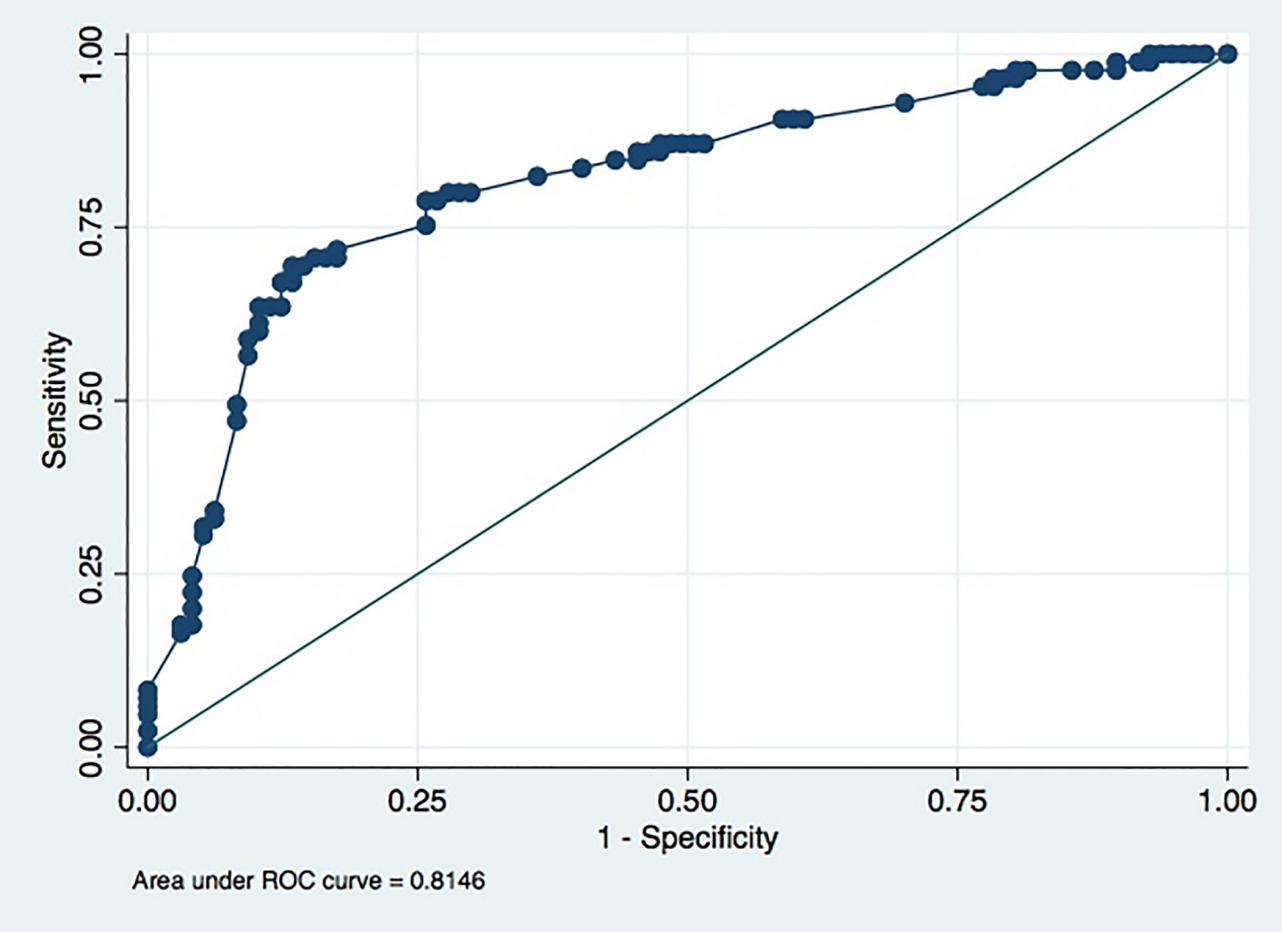
ROC curve of best model in diagnostic sensitivity and specificity of breath test, calibration phase. ROC = Receiver Operating Characteristic.

**Fig 4:**
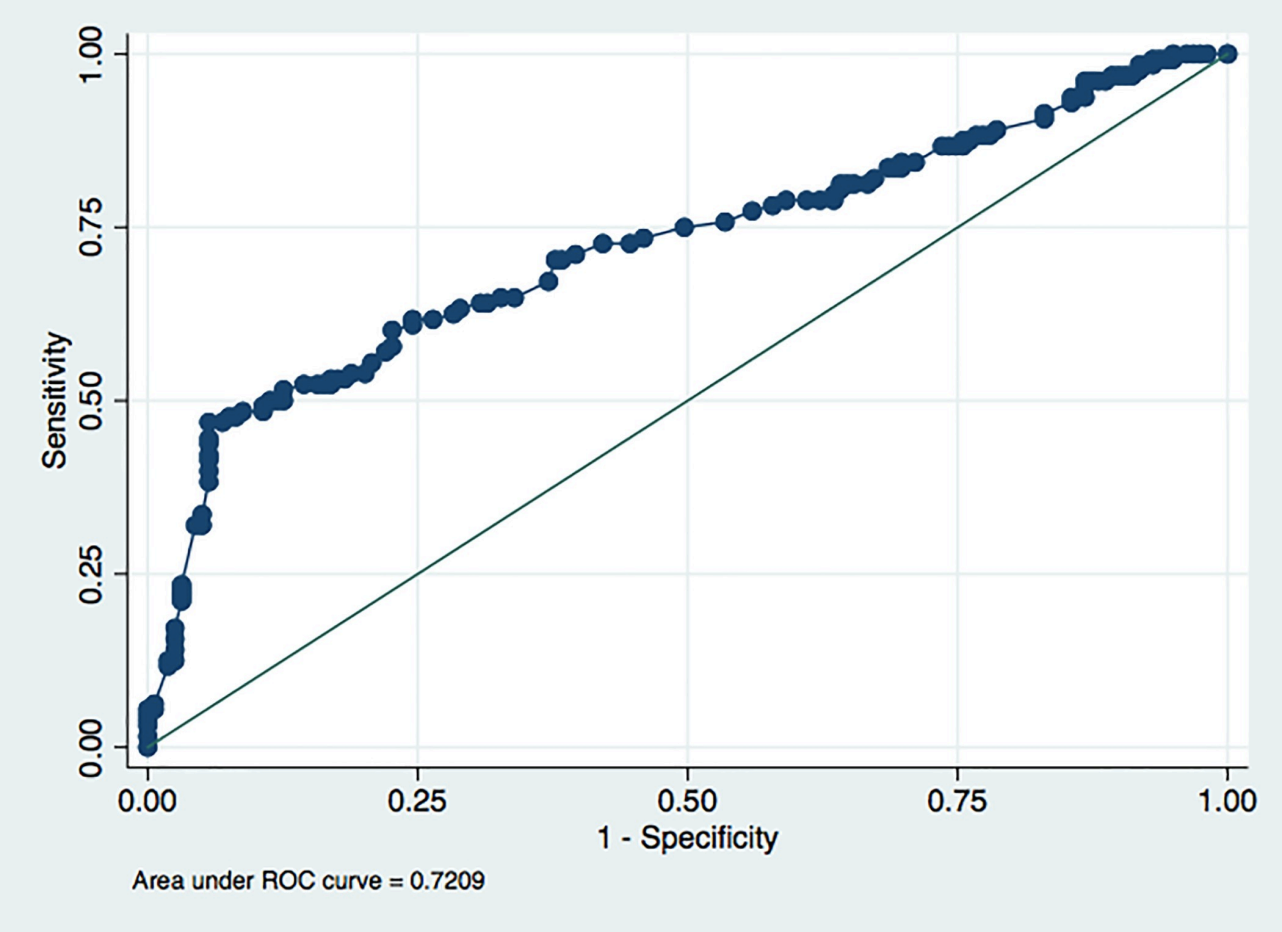
ROC curve of best model in diagnostic sensitivity and specificity of breath test, validation phase. ROC = Receiver Operating Characteristic.

**Fig 5:**
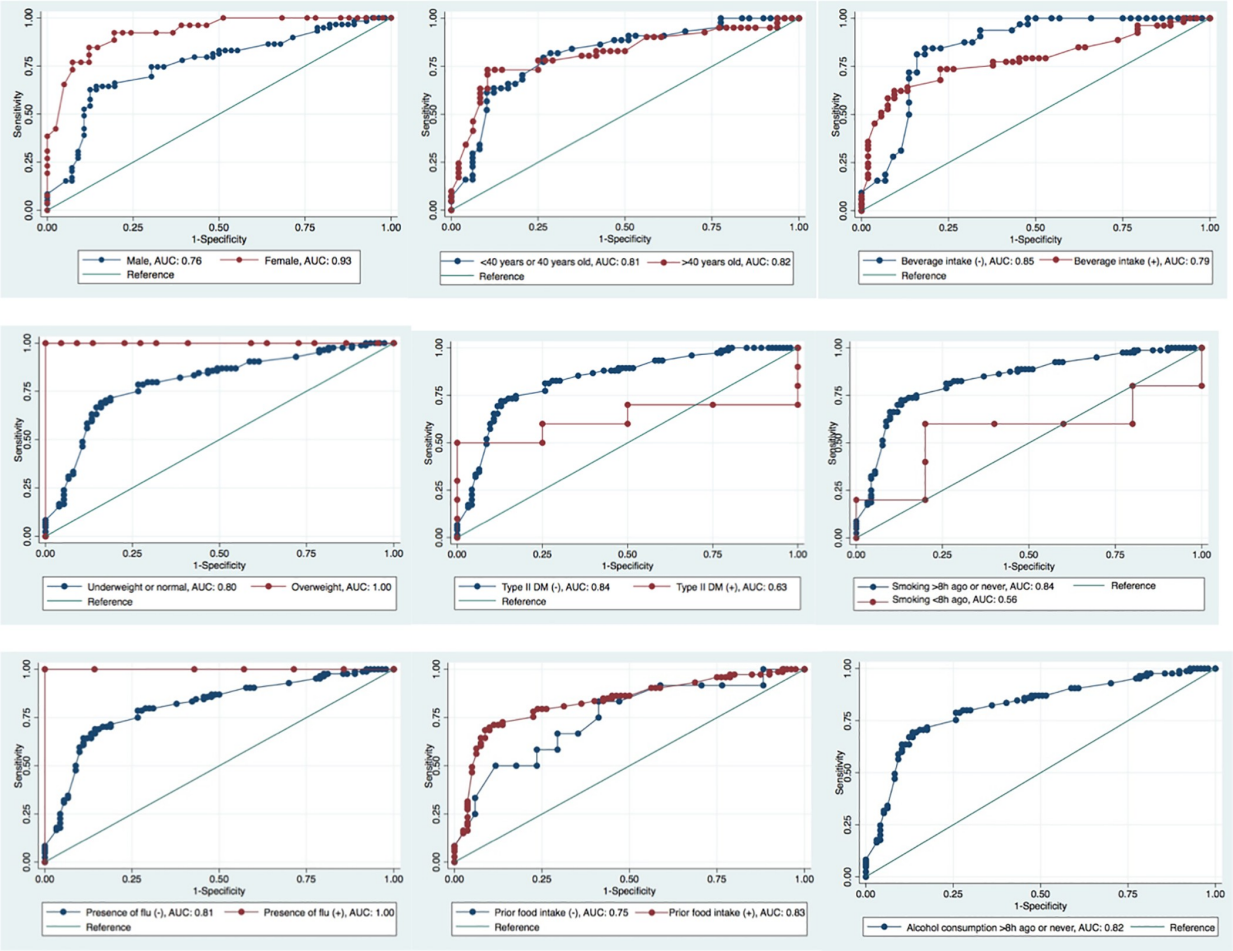
ROC curves of sensitivity and specificity of breath test in calibration phase, stratified by variables. ROC = Receiver Operating Characteristic, AUC = Area Under the Curve. ROC curves of best model in diagnostic sensitivity and specificity of breath test stratified by presence of HIV infection, COPD, asthma, or physiological stress could not be generated due to missing value of sensitivity or specificity in one strata.

Table 3 shows that in the calibration phase, the number of breath test results with false prediction of the presence of PTB was 57, while number of true breath test results was 125. Fig 3 shows the ROC curve of best model in sensitivity and specificity of breath test in the calibration phase; sensitivity was 85% (95%CI = 75–92) and specificity was 55% (95%CI = 44–65) (Table 4). ROC curves from each strata in various variables are shown in Fig 4, while Table 5 shows that the test was significantly more sensitive and specific for women than for men. Fig 5 shows the ROC curve of best model in sensitivity and specificity of breath test in the validation phase; sensitivity was 78% (95%CI = 70–85%), specificity was 42% (95%CI = 34–50%), PPV was 52% (95%CI = 48–56%), and NPV was 71% (95%CI = 62–78%) (Table 6).

**Table 3:** Examination results of study participants in each category.

Examination					
Calibration phase	Healthy Controls (n = 40)	Active PTB (n = 85)	No PTB (n = 57)		
Validation phase		Active PTB (n = 85)	No PTB (n = 57)	Probably active PTB (n = 43)	Probably no PTB (n = 102)
Symptoms					
Cough>2 weeks (yes/no), n	0/40	83/2	52/5	42/1	102/0
Unintentional ≥5% weight loss (yes/no), n	0/40	76/9	1/56	35/8	73/29
Night sweats (yes/no), n	0/40	66/19	2/55	25/18	47/55
Sputum examination					
Three sputum smear microscopy tests (negative/ positive/ unknown), n	9/0/31	2/83/0	57/0/0	20/23/0	101/1/0
Sputum culture (negative for PTB/positive for PTB/ unknown), n	9/0/31	0/85/0	57/0/0	33/10/0	97/5/0
Chest radiography (not suggestive for PTB/ suggestive for PTB), n	40/0	0/85	57/0	5/38	73/29
Follow up (not suggesting PTB/ suggesting PTB), n	40/0	0/85	57/0	1/42	102/0
Breath test results (negative/ positive PTB), n	27/13	13/72	26/31	15/28	41/61

PTB: pulmonary tuberculosis

**Table 4:** Performance of breath test results in the calibration phase.

	Final diagnosis		Sensitivity (95% CI)	Specificity (95% CI)	AUC^a^ (95% CI)
	PTB (n = 85)	No PTB (n = 97)			
Positive breath test	72	44	85 (75–92)	55 (44–65)	82 (75–88)
Negative breath test	13	53			

PTB: pulmonary tuberculosis, AUC: area under the curve, CI: confidence interval

^**a**^generated from the real value

**Table 5:** Performance of breath test results in the calibration phase, stratified by various variables.

Variable	Final Diagnosis		Sensitivity (95% CI)	Specificity (95% CI)	AUC^a^ (95% CI)	*p* value
	PTB (n = 85)	No PTB (n = 97)				
**Sex, n**						0.0023
Male						
Positive breath test	47	27	64	86	76	
Negative breath test	12	29	(51–76)	(74–94)	(67–85)	
Female						
Positive breath test	25	17	81	88	93	
Negative breath test	1	24	(61–93)	(74–96)	(87–99)	
**Age, n**						0.9683
>40 years						
Positive breath test	33	20	73	90	82	
Negative breath test	8	28	(57–86)	(77–97)	(72–91)	
≤40 years						
Positive breath test	39	24	66	84	81	
Negative breath test	5	25	(50–80)	(70–93)	(72–90)	
**Body Mass Index (BMI), n**						^b^
Overweight (BMI ≥25 kg/m^2^)						
Positive breath test	1	9	100	95	100	
Negative breath test	0	13	(2.5–100)	(77–100)	(100–100)	
Underweight or Normal (BMI<18.5 kg/m^2^ or 18.5-<25 kg/m^2^)						
Positive breath test	71	35	69	84	80	
Negative breath test	13	40	(58–79)	(74–91)	(73–87)	
**Type II diabetes mellitus, n**						0.1771
Presence of Type II DM (+)						
Positive breath test	6	2	50	75	63	
Negative breath test	4	2	(19–81)	(19–99)	(34–91)	
Presence of Type II DM (-)						
Positive breath test	66	42	72	87	84	
Negative breath test	9	51	(60–82)	(79–93)	(77–90)	
**HIV infection, n**						^b^
Presence of HIV infection (+)						
Positive breath test	1	0	100	N.A.	N.A.	
Negative breath test	0	0	(2.5–100)			
Presence of HIV infection (-)						
Positive breath test	71	44	69	87	81	
Negative breath test	13	53	(58–79)	(78–93)	(75–88)	
**Presence of COPD, n**						^b^
Presence of COPD (+)						
Positive breath test	0	1	N.A.	0	N.A.	
Negative breath test	0	0		(0–98)		
Presence of COPD (-)						
Positive breath test	72	43	69	88	82	
Negative breath test	13	53	(58–79)	(79–93)	(76–89)	
**Presence of asthma, n**						^b^
Presence of asthma (+)						
Positive breath test	1	0	0	N.A.	N.A.	
Negative breath test	0	0	(0–98)			
Presence of asthma (-)						
Positive breath test	71	44	70	87	82	
Negative breath test	13	53	(59–80)	(78–93)	(75–88)	
**Use of antibiotics, n**						0.6794
Antibiotic use (+)						
Positive breath test	7	2	75	75	86	
Negative breath test	1	2	(35–97)	(19–99)	(64–100)	
Antibiotic use (-)						
Positive breath test	65	42	69	87	81	
Negative breath test	12	51	(57–79)	(79–93)	(74–88)	
**Presence of flu, n**						^b^
Presence of flu (+)						
Positive breath test	1	3	100	100	100	
Negative breath test	0	4	(3–100)	(59–100)	(100–100)	
Presence of flu (-)						
Positive breath test	71	41	69	86	81	
Negative breath test	13	49	(58–79)	(77–92)	(74–88)	
**Presence of psychological stress due to breath test, n**						^b^
Presence of stress (+)						
Positive breath test	0	0	NA.	100	N.A.	
Negative breath test	0	2		(16–100)		
Presence of stress (-)						
Positive breath test	72	44	69	86	81	
Negative breath test	13	51	(58–79)	(78–93)	(75–88)	
**Prior food intake, n**						0.4059
Prior food intake (+)						
Positive breath test	61	34	70	90	83	
Negative breath test	12	46	(58–80)	(81–96)	(77–90)	
Prior food intake (-)						
Positive breath test	11	10	67	71	75	
Negative breath test	1	7	(35–90)	(44–90)	(56–93)	
**Prior beverage intake, n**						0.2849
Prior beverage intake (+)						
Positive breath test	41	23	60	91	79	
Negative breath test	12	30	(46–74)	(79–97)	(70–88)	
Prior beverage intake (-)						
Positive breath test	31	21	84	82	85	
Negative breath test	1	23	(67–95)	(67–92)	(77–94)	
**Smoking before test, n**						0.2082
Smoking <8 hours ago						
Positive breath test	2	1	20	80	56	
Negative breath test	3	4	(1–72)	(28–99)	(17–95)	
Smoking ≥8 hours ago or never						
Positive breath test	70	43	73	87	84	
Negative breath test	10	49	(61–82)	(78–93)	(78–90)	
**Alcohol consumption before test, n**						^b^
Alcohol consumption <8 h ago						
Positive breath test	0	0	N.A.	N.A.	N.A.	
Negative breath test	0	0				
Alcohol consumption ≥8 h ago or never						
Positive breath test	72	44	69	87	82	
Negative breath test	13	53	(58–79)	(78–93)	(75–88)	

PTB: pulmonary tuberculosis, AUC: area under the curve, CI: confidence interval

^a^generated from the real value

^b^cannot be compared because one AUC is 100% or one AUC cannot be computed.

**Table 6:** Performance of breath test results in the validation phase.

	Final diagnosis		Sensitivity (95% CI)	Specificity (95% CI)	AUC^a^ (95% CI)	PPV (95% CI)	NPV (95% CI)
	PTB (n = 128)	No PTB (n = 159)					
Positive breath test	100	92	78 (70–85)	42 (34–50)	72 (66–78)	52 (48–56)	71 (62–78)
Negative breath test	28	67					

PTB: pulmonary tuberculosis, AUC: area under the curve, CI: confidence interval, PPV: positive predictive value, NPV: negative predictive value

^a^generated from the real value

There were no adverse events (e.g., breathless, infection, or bleeding) associated with the study intervention.

## Discussion

This is the first study testing the e-nose (Aeonose) to diagnose PTB among patients with suspected PTB. The study in Bangladesh used a prototype of the e-nose (participants exhaled into a bag, then the bag content was examined using a laboratory version of the e-nose), and with smaller sample size [11]. Other studies with e-nose devices did not diagnose PTB among patients with suspected PTB [12,24,25]. The sensitivity in our study was modest, while specificity was low.

We evaluated several factors that may associate with the breath prints, *i*.*e*. physiological factors (age, sex, food, beverages), pathological and disease-related conditions (smoking, comorbidities, medication), and sampling-related issues (bias with VOCs in the environment) [26]. A previous study revealed that older age altered breath prints in patients with lung cancer [27]. Patients with high body mass index (BMI) had more false-positive test results compared to patients with normal or low BMI [12], males had higher level of isoprene compared to females [28], consuming poultry meat, plant oil, and some beverages could be differentiated by an e-nose, and smoking increased the levels of benzene and pentane [29–34]. In our study, we found that the sensitivity and specificity of the breath test were lower in men compared to women. The cause was not entirely clear; higher level of isoprene in men might reflect oxidative stress that can influence the progression of disease [35]. It could be also that a difference in smoking, eating, or other habits influences this difference. To prevent interference by VOCs in the environment such as ethanol and isopropanol [36], the Aeonose was equipped with a valve and carbon filter, thus the breath prints were not biased by the room air.

The Aeonose was developed by using arrays based on less (or non-) specific sensors combined with smart data compression and pattern recognition, namely metal-oxide sensors. This pattern recognition technique matches measured ‘patterns’ to previously ‘seen’ patterns. Therefore, a substance, or mixtures of substances, can only be recognized after a calibration phase: in order to match a pattern, it must be known beforehand (*i*.*e*., ‘seen’ before). In exhaled breath, several thousands of VOCs can be recognized. When comparing breath patterns between people suffering from a certain disease and controls, the Aeonose can be taught to differentiate and identify the diseased population. This technique provides less detailed information compared to chemical-analytic methods or spectroscopic techniques that work by detecting VOC markers, although classification of the results remains possible. Philips *et al*. found that naphthalene, 1-methyl- and cyclohexane, 1,4-dimethyl- were breath VOC markers that are sensitive and specific for pulmonary TB [37]. Detecting these markers might lead to better sensitivity and specificity. However, the insights of what VOC-markers should be picked are changing over time, resulting in other combinations of VOC-markers. The chemical-analytic methods or spectroscopic techniques also have several drawbacks: they need well-conditioned environment, especially when concentration differences of biomarkers are to be recorded; they cannot be used as a point-of-care diagnostic test due to their large size; and well-trained staff are needed to operate the devices.

The Aeonose provides non-invasive diagnosis support in minutes, is easy-to-use, without the need for robust training. In a new release of the Aeonose, an iPad is used, and the test result is provided within seconds. Charging of the device only needs a low electricity usage, as with charging of mobile phones. It is portable, thus suitable as a screening tool. Therefore, it might help to prevent TB transmission, also serve well in health care settings, with further advanced and more expensive testing for individuals picked up by the breath test.

We excluded non-TB patients with a previous history of pulmonary and extra pulmonary TB because several breath profiles of non-TB patients with a previous history of TB showed similar breath profiles with TB. Scares and persistent or dead MTB in the lung could be mimicries of TB [38]. We also excluded patients with cancer because cancer interferes with the breath test [8,9]. We were aware of the fact that in daily practices it is difficult to separate patients with lung cancer from patients with TB as patients with lung cancer are likely to have a clinical presentation that mimics TB, however, the number of patients with cancer was also too low (5 patients) precluding a separate group data analysis. The diagnostic potential if any of the Aeonose for these patients was not addressed in this study.

This study revealed lower sensitivity and specificity in comparison to the study from Bangladesh [11] and Paraguay [12], which is likely caused by the difference in the study populations. In our study, the non-PTB patients were patients with suspected PTB, thus it captured the infectious or non-infectious lung diseases, and acute or chronic lung diseases. In the study from Paraguay, non-TB subjects comprised asthma and COPD patients (patients with stable chronic lung disease), who usually have quite distinct clinical presentations than TB patients. The diversity of participants in our study is larger than in the Bangladesh and Paraguay studies, thus probably more participants are needed in the calibration phase to get comparable performance between calibration and validation phase. When an artificial neural network should predict a breath profile it hasn’t ‘seen’ during the calibration phase, a false prediction is more likely. A larger calibration group would improve the blind predictions.

Using the current sensitivity, 22% of patients would be missed, which is higher than screening by CXR which has 87% sensitivity [39]. Nevertheless, the breath test has advantages of being portable, easy-to-use, and without radiation exposure making it suitable as a repeatable screening test. Notably, approximately 30% of active tuberculosis cases are currently not detected by the national health care system [40]; as calculated within the last TB incidence [1], this test will improve case finding by as many as 67,360 cases. Furthermore, this test has higher sensitivity than symptoms screening, that only had 63% sensitivity from our data, or 70% sensitivity from a previous systematic review [39].

The strength of this study is that cohorts of patients and controls were recruited as a calibration and validation group, with the potential to confirm the correct classification. This study also has some limitations. It was performed in Yogyakarta province alone, however the organization of lung clinics in Yogyakarta is typical and representative for Indonesia. We used Löwenstein-Jensen culture, which has lower sensitivity compared to liquid culture, and we used mostly only one specimen for culture. Nevertheless, Löwenstein-Jensen culture may have higher specificity due to lower contamination rates [41–43]. As mentioned above, the e-nose provides less detailed information compared to chemical-analytic methods or spectroscopic techniques. To improve the sensitivity and specificity of the device, a larger calibration group needs to be involved. Once the VOC-markers for TB are adequately determined, the use of highly selective sensors that target these VOC-markers may also add the sensitivity and specificity.

In conclusion, the Aeonose had modest sensitivity and low specificity to diagnose TB among patients with suspected TB in Indonesia. With its portable form, it could be used for TB screening in remote rural areas with difficult access to health care facilities, as well as a screening tool in health care settings to reduce the risk of nosocomial TB transmission.

## Supporting information

S1 Appendix Enose data_supplementary materials. (XLS)
